# Development of Allosteric Ribozymes for ATP and l-Histidine Based on the R3C Ligase Ribozyme

**DOI:** 10.3390/life14040520

**Published:** 2024-04-17

**Authors:** Yuna Akatsu, Hiromi Mutsuro-Aoki, Koji Tamura

**Affiliations:** 1 Department of Biological Science and Technology, Tokyo University of Science, 6-3-1 Niijuku, Katsushika-ku, Tokyo 125-8585, Japan; 8321502@alumni.tus.ac.jp (Y.A.); haoki0930@gmail.com (H.M.-A.); 2 Research Institute for Science and Technology, Tokyo University of Science, 2641 Yamazaki, Noda, Chiba 278-8510, Japan

**Keywords:** R3C ligase ribozyme, allosteric regulation, RNA, RNA world, origin of life

## Abstract

During the evolution of the RNA, short RNAs are thought to have joined together to form long RNAs, enhancing their function as ribozymes. Previously, the artificial R3C ligase ribozyme (73 nucleotides) was successfully reduced to 46 nucleotides; however, its activity decreased significantly. Therefore, we aimed to develop allosteric ribozymes, whose activities could be regulated by effector compounds, based on the reduced R3C ligase ribozyme (R3C-A). Among the variants prepared by fusing an ATP-binding aptamer RNA with R3C-A, one mutant showed increased ligation activity in an ATP-dependent manner. Melting temperature measurements of the two RNA mutants suggested that the region around the aptamer site was stabilized by the addition of ATP. This resulted in a suitable conformation for the reaction at the ligation site. Another ribozyme was prepared by fusing R3C-A with a l-histidine-binding aptamer RNA, and the ligase activity increased with increasing l-histidine concentrations. Both ATP and l-histidine play prominent roles in current molecular biology and the interaction of RNAs and these molecules could be a key step in the evolution of the world of RNAs. Our results suggest promise in the development of general allosteric ribozymes that are independent of the type of effector molecule and provide important clues to the evolution of the RNA world.

## 1. Introduction

All life on Earth is based on the concept of “central dogma”, the unidirectional flow of genetic information [1]. When considering the evolution of genetic information transmission systems on primitive Earth, it is difficult to imagine that the complex systems of DNA, RNA, and proteins of modern life suddenly appeared. DNA is necessary for protein synthesis, and proteins are necessary to synthesize DNA and RNA. Therefore, which was generated first, nucleic acids or proteins? There is some debate as to whether “central dogma” is in fact correct in the origin of life. Biological reactions have long been thought to be controlled by enzymes and catalysts comprising proteins. An enzyme (reverse transcriptase) that synthesizes DNA using the base sequences of RNA as a template has been discovered [2], proving that RNA carries genetic information similar to that of DNA. Furthermore, the catalytic activity of RNA has been demonstrated without the involvement of proteins [3,4]. Thus, RNA has both “genetic information storage” and “catalytic” functions. The discovery of ribozymes led to the proposal of the RNA world hypothesis, which states that in the early developmental stages of life on Earth, RNA possessed both the genetic information storage function of DNA and the catalytic function of proteins; thus, the activities of living organisms were carried out solely by RNA [5].

Although modern ribosomes are a complex of RNAs and proteins, they would have assembled during early evolution by the association and joining of small functional RNA units [6]. Noller and coworkers’ experiment suggested that the peptidyl transferase center (PTC) on the ribosome is composed of RNA [7], and the crystal structure of the PTC clearly proved it [8,9]. PTC is formed by two symmetrically arranged tRNA-like units [10,11,12,13,14], and certain combinations of the symmetrical segments are capable of mediating peptide bond formation [15,16], even between two aminoacyl-minihelices (primordial tRNAs), tethered by the dimeric scaffold [16]. In the evolution of rRNA, introns may have provided the means to ligate many of these pieces together. A survey of rRNA intron sequences, locations, and structural characteristics across the three major life domains suggests that rRNA gene loci may have served as evolutionary nurseries for intron formation and diversification [17]. In addition, based on recent scientific advances in *Coronaviridae*, the transition between RNA- to DNA-based life RNA is suggested to be driven by an evolutionary relationship between RNA polymerases, RNA exonuclease, and RNA methyltransferases [18].

**Figure 1:**
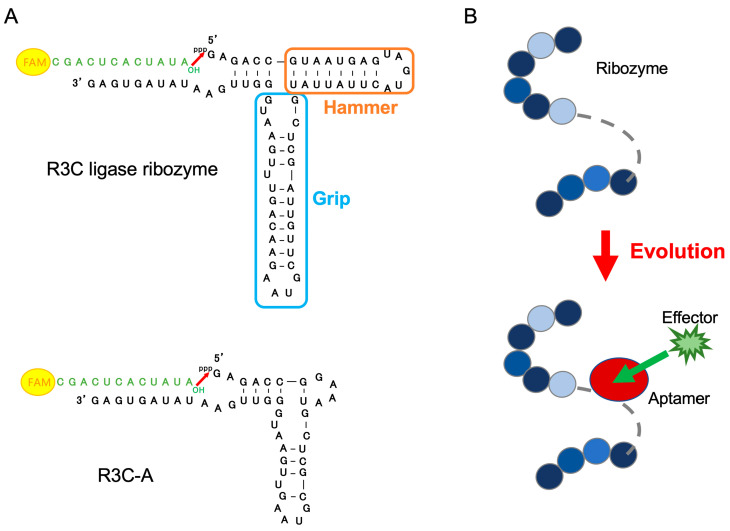
(**A**) Compositions of the R3C ligase ribozyme (73-mer, top) and that of the reduced form, R3C-A (46-mer, bottom). They are shown in black. The 5′-6-FAM-labeled ligation substrate (12-mer) is shown in green. The stem loop regions of the original R3C ligase ribozyme are designated as “Hammer” and “Grip”. (**B**) Schematic presentation of the evolution process of ribozymes. During the RNA elongation process, a short RNA would have fused with other RNA domains that interacted with specific compounds (effectors), resulting in an RNA whose activity regulation was dependent on the specific effector. Each circle represents a nucleotide comprising RNA.

It would be extremely important to conduct experiments that demonstrate an aspect of the RNA world: the concatenation of RNA domains to give rise to new functional RNAs. Ribozymes have various catalytic abilities such as cleavage, ligation, and splicing. In this study, we used the R3C ligase ribozyme (73 nucleotides [nt]) [19], which catalyzes a nucleophilic attack by a 3′-hydroxyl of the substrate on a 5′-α-phosphorus of the triphosphates of the ribozyme itself to form a 3′-5′-phosphodiester bond (Figure 1A). Eigen’s concept of a “hypercycle” argues that a length of less than 100 nucleotides is required for self-replication without necessitating an error-correcting mechanism [20]. In addition, the length of RNA synthesized in the clay mineral environment that was expected to have existed on primitive Earth is less than 50 nt [21]. Consequently, Kurihara et al. reduced the size of the R3C ligase ribozyme from 73 to 46 nt [22]. However, the ligase activity of RNA minimized to 46 nt is only 0.8% of the activity of the full-length R3C ligase ribozyme (73 nt). To restore the activity of the reduced ligase, Tanizawa et al. performed an experiment focusing on the loop portion of a minimized ribozyme [23]. RNA stabilizes its structure by forming kissing loops [24,25,26]. Using this interaction, two RNAs with complementary sequences in the loop regions of R3C ligase variants were prepared, and the mixed RNA system showed significantly increased ligation activity compared to that of the individually performed reactions. Multiple functions can be achieved via kissing loop interactions, suggesting their versatility in the evolution of RNA [27].

Allosteric regulation of protein enzymes involves allosteric binding sites, located apart from the active site of the enzyme [28]. Binding of the effector molecule to the allosteric binding site induces conformational changes in the protein structure that alter the catalytic rate of the enzyme. In the case of hemoglobin [29], the binding of oxygen to one of the subunits induces conformational changes that are relayed to the other subunits, making them more able to bind oxygen by raising their affinity for this molecule [30]. Similarly, during the evolution of the RNA world, it is probable that during the RNA elongation process, a short RNA would have fused with the domain that interacted with a specific compound (effector), resulting in an RNA whose activity was regulated by and dependent on a specific effector (Figure 1B). In the L1 ligase ribozyme [31], a ribozyme is created by fusing an ATP-binding aptamer RNA to create an allosteric ligase ribozyme that increases ligase activity in an ATP concentration-dependent manner [32]. Using the R3C ligase, which is smaller than the L1 ligase, we aimed to develop general allosteric ribozymes that are independent of the effector molecule type.

## 2. Materials and Methods

### 2.1. Preparation of R3C Ligase Ribozyme Variants and RNA Substrate

Unlabeled deoxyribonucleotides were synthesized by Eurofins Genomics K.K. (Tokyo, Japan). Each template DNA was prepared from chemically synthesized deoxyribonucleotides carrying the T7 promoter and the sequences corresponding to variants of R3C ligase ribozyme, and two synthetic primers using the polymerase chain reaction. RNA transcription was performed at 37 °C for 16 h in a reaction mixture containing 40 mM Tris-HCl (pH 8.0), 10 mM dithiothreitol, 2 mM spermidine, 8 mM MgCl_2_, 2.5 mM each NTP, template DNA (0.2 mg/mL), and pure T7 RNA polymerase (~100 µg/mL) [33,34]. The transcripts were purified by denaturing 12% polyacrylamide gel electrophoresis. The concentrations of purified RNA were determined from the UV absorbance at a wavelength of 260 nm by using Implen NanoPhotometer (München, Germany). HPLC-purified 5′-terminal 6-carboxyfluorescein (6-FAM)-labeled oligonucleotide (5′-FAM-CGACUCACUAUA-3′) was prepared by Japan Bio Services Co., Ltd. (Saitama, Japan) and was used as an RNA substrate in the ligation reaction.

### 2.2. Analysis of Ligation

Ligation analysis was performed by the method of Rogers and Joyce with a slight modification [22]. The R3C ligase ribozyme variants, dissolved in solution containing 50 mM Tris-HCl (pH 8.5) and 15 mM MgCl_2_ with effector compounds (nucleotide triphosphates, amino acids, or imidazole shown in Results) were first heated to 37 °C for 5 min and then cooled to 4 °C. Then, the ligation reaction was started by adding 0.75 µL of 100 µM substrate to the solution. The final concentration of each ribozyme and the substrate was 5 µM each. The volume of the reaction mixture was 15 µL. The concentration of the effector molecules is described in each figure legend. Except for the time course determination, after incubation at 23 °C for 18.5 h, the solution was applied to denaturing 12% polyacrylamide gel for electrophoresis. For the time course determination, after incubation at 23 °C, 10 μL of aliquots were removed at specific time points, put into 5 μL of 7 M urea and 0.08% (*w*/*v*) bromophenol blue, frozen quickly with liquid nitrogen and stored at −20 °C before electrophoresis. The gel was analyzed on a Typhoon FLA 7000 (GE Healthcare Japan, Tokyo, Japan), and the ligated products were quantified using Image Quant TL software (version 8.2.0.0).

### 2.3. UV-Monitored Thermal Denaturation Analysis

UV-monitored thermal denaturation analysis was carried out using a V-730Bio spectrophotometer (JASCO Corporation, Tokyo, Japan). Before measurements, the temperature controller of the photometer was connected to the low-temperature thermostatic water bath NCB-2500 (Tokyo Rikakikai Co., Ltd., Tokyo, Japan), and cold water was circulated at 20 °C. The UV-melting profiles of each RNA (2 µM) were measured in 50 mM Tris-HCl (pH 8.5), 15 mM MgCl_2_, and ATP (with the concentrations listed in the figures) at a scan rate of 1.0 °C/min in the range of 10–95 °C with detection at 260 nm. The first derivative was calculated from each UV-melting profile and *T*_m_ was determined.

## 3. Results

Kurihara et al. cut the stem regions of the Hammer and Grip portions of the R3C ligase ribozyme (Figure 1A) to gradually shorten the length of the entire ribozyme [22]. By creating a mutant lacking the Hammer and Grip regions, Kurihara et al. demonstrated that even ~50 nt of the R3C ligase ribozyme had ligase activity; however, its activity was reduced drastically. In the present study, we prepared a reduced mutant of the R3C ligase ribozyme (R3C-A; Figure 1A) and used it as the initial RNA.

### 3.1. Ribozyme Fused with ATP-Binding Aptamer RNA

**Figure 2:**
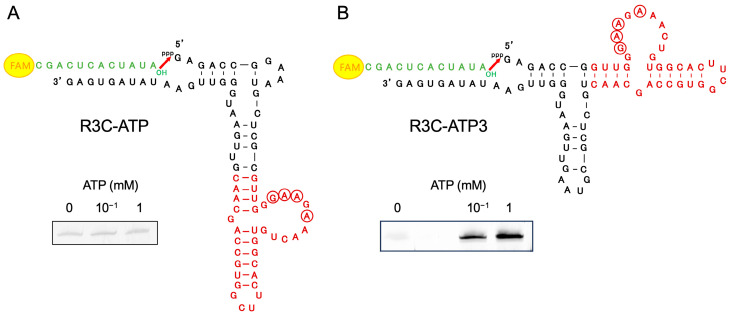
Compositions and ligation activities of (**A**) R3C-ATP and (**B**) R3C-ATP3. They were constructed by fusing an ATP-binding aptamer RNA (dark red) [35] to R3C-A. The 5′-6-FAM-labeled ligation substrate (12-mer) is shown in green. Ligation reactions were performed in varying concentrations of ATP (0 mM, 10^−1^ mM, and 1 mM). GAA in the loop, which constitutes the GNRA motif with the base of ATP as the fourth residue, and the one-letter skipped A, which forms a hydrogen bond with ATP, are circled [35].

An ATP-binding aptamer RNA [35] was fused to the Grip portion of R3C-A to produce R3C-ATP (Figure 2A). When changing the ATP concentration in the ligation reaction to 0 mM, 10^−1^ mM, and 1 mM, faint fluorescence was observed across all detected bands. Additionally, nearly no difference in fluorescence intensity was observed despite the presence or absence of ATP (Figure 2A). Therefore, R3C-ATP3 was prepared by fusing the ATP-binding aptamer RNA to the Hammer portion of R3C-A (Figure 2B), and the ligation reaction was performed by changing the ATP concentration in the reaction solution. A difference was observed in the fluorescence intensity of the bands; therefore, the higher the ATP concentration, the stronger the fluorescence (Figure 2B).

### 3.2. Ligation Reaction with Varying ATP Concentrations, and Analysis of the Effect of Other Nucleotide Triphosphates

**Figure 3:**
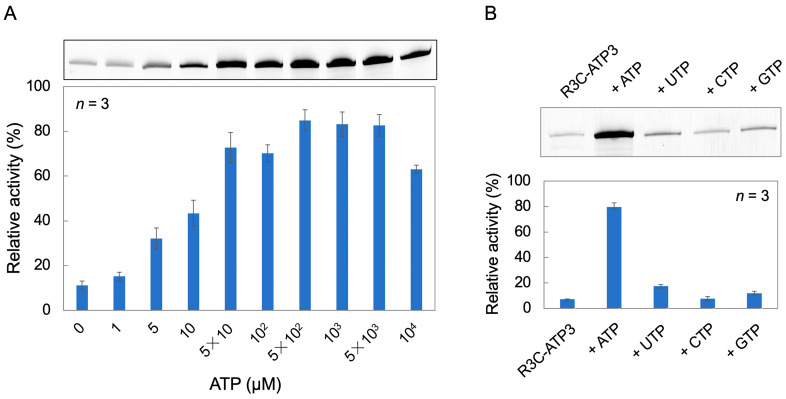
(**A**) ATP concentration dependence of R3C-ATP3 ligation activity. (**B**) Trinucleotide dependence on the ligation activity of R3C-ATP3. The final concentration of each trinucleotide is 1 mM and “R3C-ATP3” indicates the result without any trinucleotide. The activities are shown as relative values (%) compared to those in the case of the full-length R3C ligase ribozyme (100%). Error bars represent the standard deviation of triplicate experiments.

Using R3C-ATP3, the ligation reaction was performed by varying the ATP concentration from 0 to 10^4^ μM in the ligation reaction solution. The ligase activity increased as the ATP concentration increased from 0 to 5 × 10 μM. Furthermore, at ATP concentrations of 5 × 10^2^ μM, 10^3^ μM, and 5 × 10^3^ μM, the ligase activity increased to ≥80%. However, at an ATP concentration of 10^4^ μM, the activity decreased to 63% (Figure 3A).

To investigate whether the increase in ligase activity is dependent on ATP, we compared the activities of other nucleotide triphosphates. ATP, UTP, CTP, and GTP (1 mM each) were added to the ligation reaction solution and ligation reactions were performed. Ligase activity was particularly elevated in the presence of ATP, but other nucleotides resulted in little increase in activity (Figure 3B).

### 3.3. Ligation Reaction of R3C-ATP3 Mutants

**Figure 4:**
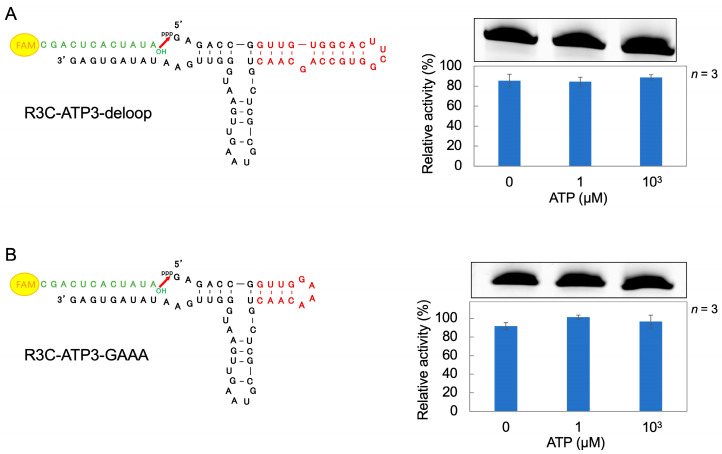
Compositions and ligation activities of (**A**) R3C-ATP3-deloop and (**B**) R3C-ATP3-GAAA. They were constructed based on R3C-ATP3 (Figure 2B). The 5′-6-FAM-labeled ligation substrate (12-mer) is shown in green. Ligation reactions were performed in varying concentrations of ATP (0 μM, 1 μM, and 10^3^ μM). The activities are shown as relative values (%) compared to those in the case of the full-length R3C ligase ribozyme (100%). Error bars represent the standard deviation of triplicate experiments.

R3C-ATP3 mutants were constructed to investigate which structure in the ATP-binding aptamer portion of R3C-ATP3 affects the ligase activity. First, we created a mutant R3C-ATP3-deloop, in which the loop structure of the ATP-binding aptamer portion was removed, and varied the ATP concentration in the reaction solution (0 μM, 1 μM, and 10^3^ μM). High ligase activity was observed across all tested ATP concentrations (Figure 4A). Next, the ATP-binding aptamer portion of R3C-ATP3 was shortened and R3C-ATP3-GAAA was prepared by adding a GAAA tetraloop. High ligase activity was observed across all tested ATP concentrations (Figure 4B).

**Figure 5:**
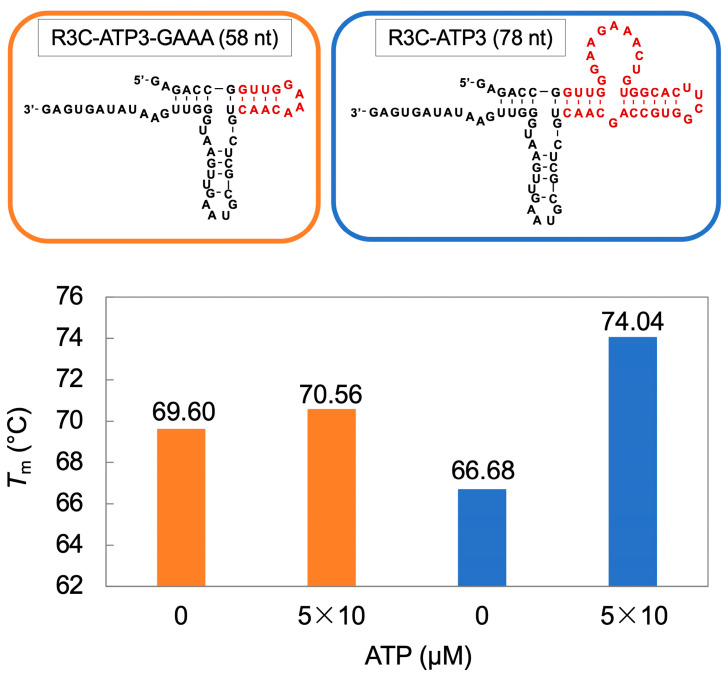
UV-monitored thermal denaturation analysis for R3C-ATP3-GAAA (orange) and R3C-ATP3 (blue) in the presence or absence of ATP. *T*_m_ values were assessed by determining the UV absorbance at 260 nm. The dark red region indicates the ATP-binding aptamer derived portion.

The *T*_m_ value serves as an indicator of RNA stability. Ligase activity changes with variations in ATP concentration; therefore, we hypothesized that the presence or absence of ATP may cause changes in the structure of the ATP-binding aptamer RNA portion of the ribozyme and attempted to measure the *T*_m_ value. In R3C-ATP3 and R3C-ATP3-GAAA, the ATP concentration was changed to 0 μM and 5 × 10 μM, and the temperature change measurement of absorbance was conducted. The *T*_m_ value was higher at 5 × 10 μM than at 0 μM. In contrast, the change in the *T*_m_ value of R3C-ATP3-GAAA was minimal (Figure 5).

### 3.4. Ribozyme Fused with l-Histidine (His)-Linked Aptamer RNA

**Figure 6:**
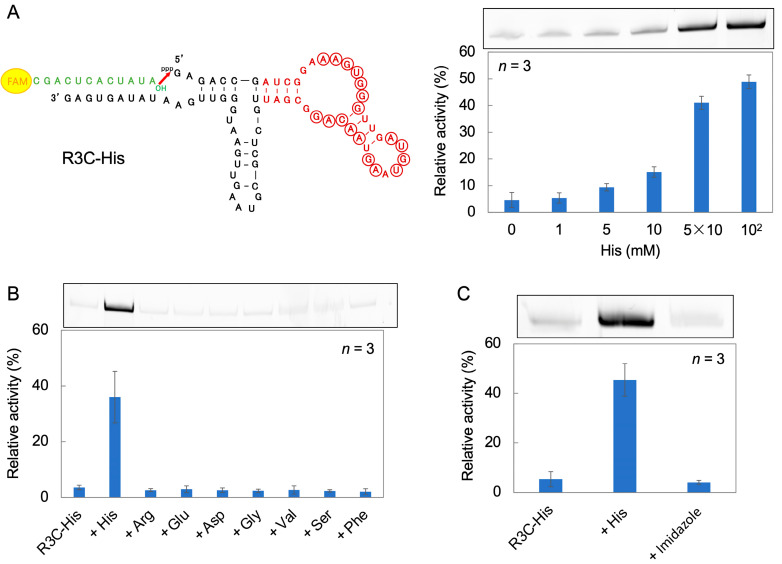
(**A**) Composition and ligation activities of R3C-His. It was constructed by fusing a His-binding aptamer RNA (dark red) [36] to R3C-A. The 5′-6-FAM-labeled ligation substrate (12-mer) is shown in green. Ligation reactions were performed in varying concentrations of His. Circled nucleotides are >62% conserved in the original selection pool [36]. (**B**) Amino acid dependence on the ligation activities of R3C-His. The final concentration of each amino acid is 5 × 10 mM, and “R3C-His” indicates the result without amino acid. (**C**) Effect of His and imidazole on the ligation activity of R3C-His. The final concentration of His or imidazole is 10^2^ mM, and “R3C-His” indicates the result without His or imidazole. The activities are shown as relative values (%) compared to those in the case of the full-length R3C ligase ribozyme (100%). Error bars represent the standard deviation of triplicate experiments.

R3C-His was prepared by fusing a His-binding aptamer RNA [36] to the Hammer portion of R3C-A. The ligation reaction was performed by varying the His concentration in the reaction solution from 0 to 10^2^ mM. Ligase activity increased as the His concentration increased (Figure 6A).

To examine the ligase activity when amino acids other than His are added, eight amino acids (l-arginine (Arg), l-glutamic acid (Glu), l-aspartic acid (Asp), glycine (Gly), l-valine (Val), l-serine (Ser), and l-phenylalanine (Phe)) were each added to the reaction solution (5 × 10 mM of each) instead of His. The ligase activity increased only in the presence of His (Figure 6B).

His is an amino acid that contains an imidazole group. To investigate the effect of imidazole groups on the ligation reaction, 10^2^ mM His or imidazole was added to the ligation reaction solution. The ligase activity increased in the presence of His, but did not change in the presence of imidazole (Figure 6C).

### 3.5. Time Course of R3C-ATP3 and R3C-His

**Figure 7:**
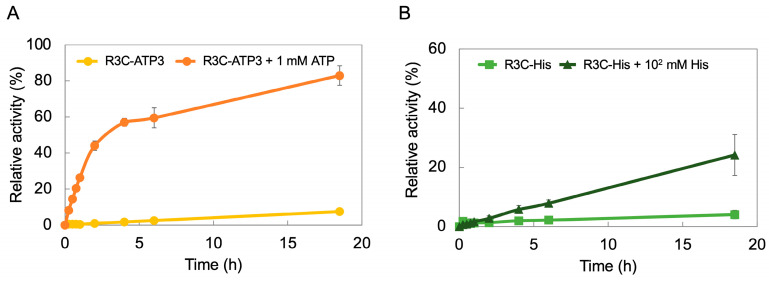
Time courses of the ligation activities of (**A**) R3C-ATP3 and (**B**) R3C-His in the presence or absence of each effector molecule. The activities are shown as relative values (%) compared to those in the case of the full-length R3C ligase ribozyme (100%). Error bars represent the standard deviation of triplicate experiments.

To examine the time course of ligase activity in the presence or absence of effector compounds, samples were tested both with and without the addition of 1 mM ATP to R3C-ATP3, and with and without the addition of 10^2^ mM His to R3C-His. A ligation reaction was performed with the samples, which was stopped at 15 min, 30 min, 45 min, 1 h, 2 h, 4 h, 6 h, and 18.5 h, respectively, after which, the ligase activity was evaluated. The activity of R3C-ATP3 was low even after 18.5 h. In contrast, when 1 mM ATP was added to R3C-ATP3, 40% activity was observed after 2 h, and 80% after 18.5 h (Figure 7A). The activity of R3C-His was low even after 18.5 h. However, when 10^2^ mM His was added to R3C-His, the activity gradually increased with the lapse in reaction time (Figure 7B).

## 4. Discussion

In contrast to R3C-ATP (in which the ATP-binding aptamer RNA was fused to the Grip portion of R3C-A (Figure 2A), R3C-ATP3 (in which the ATP-binding aptamer RNA was fused to the Hammer part of R3C-A) showed stronger fluorescence as the ATP concentration increased (Figure 2B). This suggests that ATP acts as an effector compound and contributes to an increase in the ligase activity of R3C-ATP3 but not of R3C-ATP. Although it is not apparent that the effect of grafting the aptamer is related to the proximity of the graft to the three-way junction, the graft starts right at the junction of the Hammer part in R3C-ATP3, whereas its position is distal from the junction of the Grip part in R3C-ATP. Clearly, it means that the Grip is better structured than the Hammer even without the addition of ATP. This analysis is actually well supported by the behaviors of the constructs where a rather short hairpin in the Hammer domain is sufficient to promote efficient ligation (Figure 4).

When assessing the ligase activity in the presence of different nucleotide triphosphates, we noted that the ligase activity of R3C-ATP3 changed in an ATP-dependent manner (Figure 3B). However, a minimal increase in activity was observed when adding UTP and GTP (Figure 3B). This suggests that UTP and GTP also interact with the ATP-bound aptamer RNA and ligate to the substrate.

**Figure 8:**
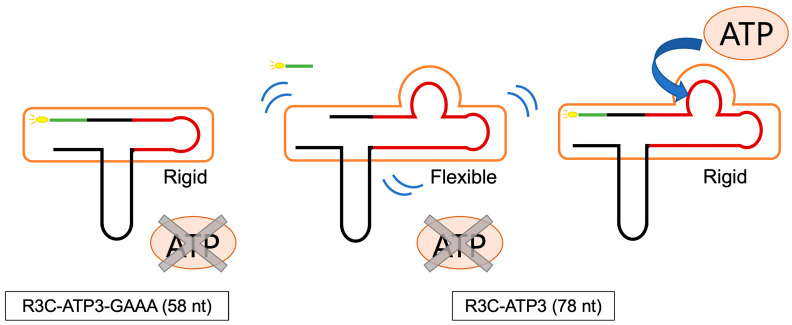
Schematic presentation of the stability of R3C-ATP3-GAAA and R3C-ATP3. The black and green lines represent the R3C-A derived portion and substrate RNA, respectively. The dark red line represents the ATP-binding aptamer derived portion. In the case of R3C-ATP3-GAAA, the stem structure around the ligation site is likely to be rigid regardless of the presence or absence of ATP. In contrast, R3C-ATP3 has a flexible conformation in the absence of ATP, and becomes stable in the presence of ATP.

R3C-ATP-deloop (in which the loop structure of the ATP-binding aptamer RNA was removed) and R3C-ATP3-GAAA (in which the structure of the ATP-binding aptamer RNA was shortened and the GAAA tetraloop structure was added) demonstrated high ligase activities and the activities did not vary with distinct ATP concentrations (Figure 4). The structural change derived from the loop structure within the ATP-binding aptamer RNA is believed to be important for facilitating its interaction with ATP and modulating the activity of the ribozyme. The three-dimensional structure of the AMP-bound form of the ATP-binding aptamer RNA has been solved by NMR spectroscopy, and the GAA of the loop structure with the AMP forms a GNRA motif [35]. A similar motif may also be formed in R3C-ATP3, and this may be related to changes in the ligase activity. The results for R3C-ATP3-GAAA indicate that the stability of the stem structure may be involved in this activity. The measurement of the *T*_m_ value also suggests that the stem structure of R3C-ATP3 becomes rigid (stable) in the presence of ATP (Figure 5). Thus, structural rigidity and flexibility affect ligase activity (Figure 8).

There are two known classes of bis-(3′-5′)-cyclic dimeric guanosine monophosphate (c-di-GMP)-binding riboswitches, which bind c-di-GMP and regulate the expression of genes in response to binding to this c-di-GMP [37,38]. The crystal structures of these c-di-GMP riboswitch aptamers show that the ligand binds within a three-helix junction that involves base-pairing and extensive base-stacking strengthen the stability of the structure [39,40,41]. Our present results also remind us of the conformational characteristics of modified uridines in the first letter of the anticodons (34th position) of tRNAs. Derivatives of 5-hydroxyuridine (xo^5^U) are much more “flexible” than unmodified uridines, whereas 5-methyl-2-thiouridine (xm^5^s^2^U) derivatives are much more “rigid” than unmodified uridines. The xm^5^s^2^U is retained in the usual C3′-endo form and is allowed to form a stable base pair with adenosine, as the third letter of the codons, but never with uridine. In contrast, xo^5^U takes the unusual C2′-endo form as well as the usual C3′-endo form. It therefore forms base pairs with uridine and guanosine as the third letter of the codons and forms standard base pairs with adenosine. Thus, such post-transcriptional modifications of U at the 34th position of tRNA allow for the correct and efficient translation of codons in protein biosynthesis [42].

Similar to R3C-ATP3, R3C-His (which was prepared by fusing a His-binding aptamer to the Hammer portion of R3C-A) showed His-dependent ligation activity (Figure 6). This suggests that His also acts as an effector compound and contributes to the increase in R3C-His ligase activity. When comparing ligase activity in the presence of eight amino acids, we observed that ligase activity increased solely in the presence of His (Figure 6B) and that R3C-His ligase activity was modulated in a His-specific manner. However, the whole amino acid structure of His may be involved in this activity because imidazole, the side chain of His, did not affect ligase activity (Figure 6C).

Thus, we successfully created allosteric ribozymes based on the R3C ligase by adding the specific domains for interacting different effector compounds (ATP and His). ATP is one of the four nucleotide monomers used for RNA synthesis and provides energy to drive and support many processes in living cells [43]. On the other hand, His is biosynthesized from ribose 5-phosphate and ATP [44]. Furthermore, His plays a prominent role in the acid–base chemistry of many enzymes and is the most common amino acid found in the active site of enzymes [45]. Considering these facts, the interaction of RNAs and these molecules could be a key step in the evolution of the RNA world, including the origin of the genetic code. In the process of the evolution of ribozymes, similarly in the case of protein enzymes, enzymatic processes subject to allosteric regulation could be important. The “hypercycle” is a cooperative self-reproducing system composed of RNAs and enzymes [20]. The cyclic behavior enhances the stability of the system, and the replication accuracy is increased. Before the establishment of such a hypercycle self-replicating system, allosteric roles appended by the effector-binding domains may have functioned instead of independent enzymes.

In the most general sense, domain–domain communication both in ribozymes and protein enzymes should be seen by the non-covalent assembly of the component molecules [46]. Therefore, in the case of protein enzymes, separate polypeptides should properly associate at an interface to create an active structure. However, in fact, many of the efficient reactions require, at a minimum, communication that depends on covalent continuity in protein enzymes [46], which would also apply to ribozymes.

Crystal structures of proteins elucidate that many proteins consist of several domains within the same polypeptide, each forming a compactly folded three-dimensional structure. Similar domains appear in a variety of different functional proteins [47,48]. Thus, by comparing the status of domain arrangements, domains are used as a measure of molecular evolution. As small molecules were ligated and formed larger structures, independently existed small domain with a certain function would have been fused into an existing molecule. In the process of molecular evolution, it is important to increase the efficiency as well as the specificity of the reaction; so, in this sense, it would make sense that a form of molecular fusion via covalent bonding is used to bring two reaction sites into physical proximity. However, in earlier processes, there must have always been a phase of non-covalent intermolecular interaction by small molecules, albeit of very low activity. To clarify, this is one of the major challenges of molecular evolution.

Domain addition has a crucial significance in the evolution of biomolecules. The current protein synthesis system shows that this is not the case. In the evolution of aminoacyl tRNA synthetases (aaRSs) and its relation to tRNAs, the minihelix region (half domain of tRNA with the amino acid attachment site) would have originated first, by interacting with the conserved domain of aaRSs for amino acid activation. Then, the other tRNA half would have added later, by interacting with the non-conserved domain of aaRSs for specific recognition of an anticodon [14,49,50,51]. Similarly, in the evolution of ribosomes and its relation to tRNAs, the CCA end of the minihelix would have originated first, by interacting with the large ribosomal subunit for peptide bond formation, and the end of the other tRNA half would have added, by interacting with the small ribosomal subunit for decoding mRNA triplets through codon–anticodon interactions.

## 5. Conclusions

A ribozyme, formed by fusing an ATP-binding aptamer RNA to the Hammer portion of R3C-A (a minimized RNA mutant), exhibited ATP concentration-dependent changes in ligase activity. However, no change in ligase activity was observed in mutants with an altered ATP-binding aptamer RNA portion of R3C-ATP3 (R3C-ATP3-deloop, in which the loop structure was removed). This indicates that the loop structure is contained within the ATP-binding aptamer RNA. ATP increases the stability of R3C-ATP3 and forms a structure suitable for ligation. Moreover, a ribozyme with His-dependent activity was obtained by fusing a His-binding aptamer RNA to R3C-A. Thus, we developed general allosteric ribozymes that are independent of the type of effector molecule, providing important clues to the evolution of the RNA world.

## Data Availability

The data presented in this study are available from the corresponding authors upon reasonable request.
